# Unique contributors of contextual stress to Black mothers’ sleep in the postpartum period

**DOI:** 10.1093/sleep/zsaf106

**Published:** 2025-04-18

**Authors:** Redson Machongo, Jennifer J Doering

**Affiliations:** University of Wisconsin-Milwaukee School of Nursing, Milwaukee, WI, USA; University of Wisconsin-Milwaukee School of Nursing, Milwaukee, WI, USA

First-time postpartum mothers face a myriad of challenges that can disrupt sleep [1]. These challenges may include infant feeding and sleeping schedule, worry about the infant, depression, anxiety, balancing work and personal life, adjusting to new parental roles, relationship stress, and the physical recovery from birth [2–4]. Controlling for covariates (including infant sleep), the current study sought to understand the impact of two contextual stressors; racial discrimination and financial strain, on postpartum sleep in first-time Black mothers [5]. Given that such contextual factors are not often investigated, this is a strength of the current study.

Racial disparities in sleep characteristics exist within the adult population [6]. Research has not adequately addressed the impact of racism on maternal health and sleep patterns [1, 5, 7]. Hart and colleagues [5] explore how contextual stress measured at 1 week postpartum affects the overall health of Black mothers across the postpartum period. The study acknowledges that racial health inequities are primarily linked to the effects of racism that persist across structures, systems, and, within individual interactions. Relatedly, recent studies have documented associations between aspects of institutional racism and adverse birth outcomes [4], including postpartum morbidity and mortality among Black/African Americans as compared to white women or women of any other racial or ethnic group [5, 6, 8, 9].

Sleep is a fundamental pillar for optimal cognitive function, physiological processes, emotional regulation, and overall quality of life [10, 11]. Studies have identified sleep in the postpartum period as a significant factor contributing to health disparities between Black mothers and their white counterparts [5, 12]. Recent randomized control trials have consistently highlighted significant deviations in routine postpartum sleep patterns among new Black mothers [2–4]. Additionally, sleep health disparities are likely attributable to longstanding exposure to psychosocial stressors such as racial/ethnic discrimination, racism, and socio-demographics [1, 6].

Optimal nighttime adult sleep spans a duration of 7 to 9 h and sleep efficiency of ≥ 85% [6, 13], yet studies reveal that postpartum women may be challenged to meet these parameters. A prospective cohort study (*n* = 134 with 37% Black, 63% white) reported varying findings on short sleep experienced by both Black and white participants at 6-8 weeks postpartum [6]. At 4 to 12 months of postpartum, Black women consistently experienced shorter sleep duration, averaging 6.1–6.4 h per night, while white women exhibited an average sleep duration of 7.0–7.1 h per night during the same period [6]. Furthermore, a similar study that examined both subjective and objective sleep in low-income postpartum women (*n* = 183, 72% African-American) found that a total nighttime sleep measured by actigraphy showed no improvement from week 4 (5.5 h) to week 8 (5.4 h), and subjectively, almost 85% of the sample met criteria for poor sleep quality at week 4 [14]. While additional studies are needed, evidence suggests that the sleep trajectory for Black women may not demonstrate a similar rate of return to normal adult parameters as white women after birth, thus interventions that can close this gap are needed.

The current study utilized data from a randomized controlled trial of a responsive parenting intervention designed to promote infant sleeping and self-soothing strategies to prevent excessive weight gain among first-born Black American infants during the 16 weeks after birth [5]. A sample of 212 mothers (mean age 23 years) participated in the Sleep-Strong African-American Families project at Augusta University Medical Center in Augusta, Georgia. Notably, the intervention did not explicitly address mothers’ racial discrimination or financial strain, which may have influenced the observed outcomes. Data collection took place within participant homes at postpartum weeks 1, 8, and 16 with the intervention delivered at weeks 3 and 8 weeks [5].

Hart *et al*. [5] examined associations between racial discrimination and financial strain and mothers’ self-reported and actigraph-estimated sleep using multivariate linear regressions. Both predictors (i.e. racial discrimination and financial strain) were entered into the same model to examine their unique effects. Separate models were run for each of the five sleep outcomes. The authors controlled for infant-related factors and found that systemic racial discrimination and financial strain as social determinants of health contribute to sleep difficulties in Black postpartum women [5]. Similar findings from randomized controls showed that Black mothers face numerous risk factors due to systemic racism, including increased likelihood of material hardships, inadequate medical care, and discrimination in daily life and in medical settings [2–4]. Moreover, Black mothers are likely to experience postpartum morbidity and are twice as likely to develop postpartum complications such as severe postpartum depression compared to other racial/ethnic groups [2, 7]. The interrelatedness of these factors to sleep is necessary to take into consideration within research study designs.

There are challenges with conducting sleep research during the often overwhelming, chaotic, and sensitive nature of the early postpartum period when research may be perceived by families as intrusive [2, 15]. Hart *et al*. [5] overcame such challenges to collect data in homes at 1 week postpartum and demonstrated minimal data loss over the study period. Actigraphy offers many advantages for the study of postpartum sleep, because it possesses greater ecological validity by more accurately capturing the authentic sleep experience that transpires within an individual’s unique home environment [16]. The simultaneous use of both subjective and objective measures is generally recommended and constitutes a study strength. However, it is important to acknowledge that actigraphy and subjective sleep measures harness disparate aspects of sleep [17]. Looking at the overall study methods, one critique of the Hart *et al*. [5] study is its use of a sleep questionnaire focused on insomnia symptoms rather than overall sleep quality. Furthermore, infant sleep was measured at 8 and 16 weeks using a single item from the Brief Infant Questionnaire rather than the whole tool. A more comprehensive measure of a concept generally contributes more understanding than a single-item measure provided the participant impact is reasonable.

Structural, social, and environmental factors, including discrimination, racism, and socio-demographics factors are deeply rooted contributors to many health disparities and can be difficult to change [6, 7, 9]. However, sleep is a modifiable behavior that presents unique opportunities for innovative interventions to improve sleep equity provided social determinants of health are also addressed when designing, implementing, and evaluating such interventions.
